# Linking depressive symptom dimensions to cerebellar subregion volumes in later life

**DOI:** 10.1038/s41398-020-00883-6

**Published:** 2020-06-19

**Authors:** Hannah R. Bogoian, Tricia Z. King, Jessica A. Turner, Eric S. Semmel, Vonetta M. Dotson

**Affiliations:** 1 Department of Psychology, Georgia State University, Atlanta, GA USA; 2 Neuroscience Institute, Georgia State University, Atlanta, GA USA; 3 Gerontology Institute, Georgia State University, Atlanta, GA USA

**Keywords:** Neuroscience, Psychology

## Abstract

The present study examined the relationship between subthreshold depressive symptoms and gray matter volume in subregions of the posterior cerebellum. Structural magnetic resonance imaging data from 38 adults aged 51 to 80 years were analyzed along with participants’ responses to the Center for Epidemiologic Studies Depression Scale. Subscale scores for depressed mood, somatic symptoms, and lack of positive affect were calculated, and multiple regression analyses were used to examine the relationship between symptom dimensions and cerebellar volumes. Greater total depressive symptoms and greater somatic symptoms of depression were significantly related to larger volumes of vermis VI, a region within the salience network, which is altered in depression. Exploratory analyses revealed that higher scores on the lack of positive affect subscale were related to larger vermis VIII volumes. These results support that depressive symptom profiles have unique relationships within the cerebellum that may be important as the field move towards targeted treatment approaches for depression.

## Introduction

There is an increasing movement towards a spectrum-based view of psychopathology, reflected in the National Institute of Mental Health’s Research Domain Criteria^1^. Within this framework, mild, subthreshold depressive symptoms (SDS) are important to examine given that they represent a less severe form of depression that is still associated with negative outcomes, especially among older adults^2,3^. At least 10–13.8% of all older adults are estimated to experience SDS in late life^4,5^, which puts them at a higher risk of functional impairment^3^ and mortality compared to their healthy peers^6^. While not meeting criteria for major depressive disorder (MDD), increasing severity of SDS is nonetheless associated with brain abnormalities among older adults such as smaller right parahippocampal volumes^7^, increased cortical thickness in the isthmus cingulate and middle frontal gyrus^8^, and gray matter volume reductions in the frontal and temporal lobes^9–11^.

Symptom dimensions of depression (e.g., negative mood, somatic symptoms, and anhedonia) have also been linked to distinct structural differences in the brain^12,13^ and unique profiles of cognitive deficits among older adults^14^, and have been shown to predict treatment outcomes in individuals with MDD^15^. We previously demonstrated distinct relationships between cingulate subregion volumes and symptom dimensions of depression^16^. The present study expanded this framework to the cerebellum, which has recently been implicated in studies of emotion processing^17,18^ in addition to its well-known role in motor coordination. Habas et al.^19^ provided evidence that distinct regions of the cerebellum participate in two major networks involved in depression—the default mode (lobule IX) and salience networks (lobule VI). Further, they propose that the vermis of lobule VI could play a modulatory role in the salience network, perhaps representing a “phylogenetically older cerebellar emotional processor” ^18^ in conjunction with other areas of the posterior vermis. In further support of the affective role of the cerebellum, the vermal region of areas VIIB, VIII, and IX have connections with the amygdala^20,21^. In fact, a new intrinsically connected “cerebello-amygdaloid” network has recently been described, with a hypothesized function for bottom-up sensory processing involving emotional evaluation, motivational appraisal, and motor preparation.^22^

In an expansion of the cerebellum’s known role as a modulator of emotion processing, structural imaging studies have shown increased volume of cerebellar area IX in both acute and remitted MDD^23^. In contrast, most earlier studies showed that depressive symptoms are related to smaller vermis volumes^24^ as well as smaller overall cerebellar volumes^25,26^. These studies generally implicate posterior regions of the cerebellum, particularly the vermis^27^, with greater evidence for decreases in cerebellar volumes in depression.

Studies to date have focused on the relationship between cerebellar volume and total depressive symptoms, therefore it is not clear whether different symptom dimensions have unique relationships with subregions of the cerebellum. Accordingly, the present study examined the relationship between symptom dimensions of depression and volumes of cerebellar subregions in a population of late middle-aged and older adults with SDS. We hypothesized that overall symptom severity would be negatively associated with the gray matter volumes of vermal regions VI and VII, and that symptom dimensions would disparately predict the structural volumes of vermal regions VI and VII.

## Materials and methods

### Participants

Participants included 51 community-dwelling healthy adults aged 51–80 years. All participants were native English speakers, right-handed, had normal or corrected-to-normal vision, had nine or more years of education, and provided written and verbal consent to participate in this study. Exclusion criteria included evidence of dementia per the Telephone Interview for Cognitive Status^28^ or self-report of major or unstable medical conditions, neurological disorders including seizure and stroke, learning disorders, current use of antiepileptic or antipsychotic medication, and magnetic resonance imaging (MRI) contraindications. Additionally, any participants who met criteria for a major psychological disorder as measured by the Structured Clinical Interview for DSM-IV Axis I Disorders (SCID)^29^ were excluded from the study. Of the 51 participants, six individuals were excluded from analyses because of missing data, two were excluded due to outlier Center for Epidemiologic Studies Depression (CES-D) scores (>3 SDs above the mean), and five participants were excluded due to poor image processing quality, leaving a total sample size of 38 individuals. The research was approved by the local institutional review board. Demographic data for this sample are provided in Table 1.

**Table 1:** Sample characteristics.

	Mean	SD	Range
Age (years)	67.92	6.94	51–80
Education (years)	15.32	2.64	10–20
Sex (% female)	73.7	–	–
Ethnicity			
African–American (%)	5	–	–
Caucasian (%)	95	–	–
CES-D total score	5.50	4.97	0–19
CES-D subscales			
Depressed mood	0.84	1.44	0–6
Somatic	1.97	2.41	0–10
Lack of positive affect	1.47	2.08	0–9
Interpersonal difficulties^1^	1.21	1.68	0–6
Medical comorbidities			
Hypertension (%)	39.5	–	–
Hyperlipidemia (%)	21.1	–	–
Diabetes (%)	0	–	–
Any cardiovascular risk (%)^2^	81.6		

*CES-D* Center for epidemiologic studies depression scale.

^1^Due to limited distribution of scores, this subscale was not included in the analyses.

^2^Includes the presence of smoking, hypertension, hyperlipidemia, diabetes, heart disease, or body mass index ≥ 25.0.

### Assessment of depressive symptoms

To measure depressive symptoms, participants completed the CES-D, a commonly used 20-item self-report measure of depressive symptoms with high internal consistency among the general population (Cronbach’s *α* = 0.85)^30^. This scale was chosen for its well-documented validity in an older adult population^31–33^ in addition to its well-replicated four-factor structure, which includes depressed mood, somatic symptoms, lack of positive affect, and interpersonal difficulties^30,34,35^. Thus, the CES-D allows us to take a dimensional approach to assessing depressive symptoms. Continuous measures of CES-D total and subscale scores were used in statistical analyses. The interpersonal problems subscale was not used in the current analyses due to the limited distribution of scores in this sample (most scores were 0).

### Imaging procedures

MRI data were acquired on a Philips (Amsterdam, The Netherlands) 3-T scanner using an 8-channel head coil. Structural images were acquired using a T1-weighted turbo field echo high-resolution three-dimensional anatomical scan with 170 1-mm slices in sagittal orientation (repetition time = 28.1 ms; echo time = 3.7 ms; flip angle = 8°). Foam padding was used within the head coil to minimize motion within the scanner.

Pre-processing of the images was conducted using Statistical Parameter Mapping (SPM) Version 8 in MATLAB. Preprocessing included conversion of each image from DICOM to NIfTI files using MRICron, and then manual inspection of images for quality using FSL View. Image isolation, normalization, and segmentation were performed using the Spatially Unbiased Infratentorial Template (SUIT) Toolbox, which operates within SPM Version 8 in MATLAB^36^. The SUIT toolbox is available to download freely online (http://www.diedrichsenlab.org/imaging/suit_download.htm). Briefly, processing included motion correction, skull stripping, and segmentation of the gray and white matter tissue. Once the images were segmented, a high-resolution, spatially unbiased atlas template of the cerebellum and brainstem within SUIT was used to define regions of interest (ROI) within the cerebellar lobules^36–39^. Analyses focused on vermal regions VI and VII given that these posterior cerebellar regions have been most consistently implicated in emotion processing studies to date^18,27^.

### Statistical analyses

Multiple regression analyses were conducted using SPSS Version 24.0^40^; variables met the assumptions of regression analyses. First, CES-D scores predicted the ratio of cerebellar ROI volume to total intracranial volume, controlling for sex and age. Separate analyses were conducted for vermal regions VI and VII. Second, parallel models were conducted with the CES-D depressed mood, somatic symptoms, and lack of positive affect subscale scores as simultaneous predictors. This resulted in a total of four regression models for our primary analyses. An alpha ≤ 0.05 (two-sided) was considered significant. Power was not calculated prior to study analyses to determine sample size; however, we report effect sizes obtained with our sample of 38. The squared semi-partial correlation coefficient (sr_i_^2^) was included as a measure of effect size, given that the sr^2^_i_ coefficient indicates each predictor variable’s unique contribution to *R*^2^. In other words, sr^2^_i_ indicates the unique proportion of the outcome variable (cerebellar ROI) that is accounted for by a predictor variable (total CES-D score or a subscale score of the CES-D), beyond what is accounted for by the other predictor variables included in the regression model^41^.

Parallel exploratory models examined other cerebellar subregions as dependent variables. Vermal lobules VIII, IX, and crus I are regions of the posterior cerebellum that have also been implicated in studies of emotion processing^17,42^, but with less consistency than the subregions selected for our primary analyses. The anterior cerebellum (sum of volume in lobules I–IV) functioned as a control region given that it was not anticipated to have a relationship with depressive symptoms.

## Results

### Primary analyses

**Fig. 1:**
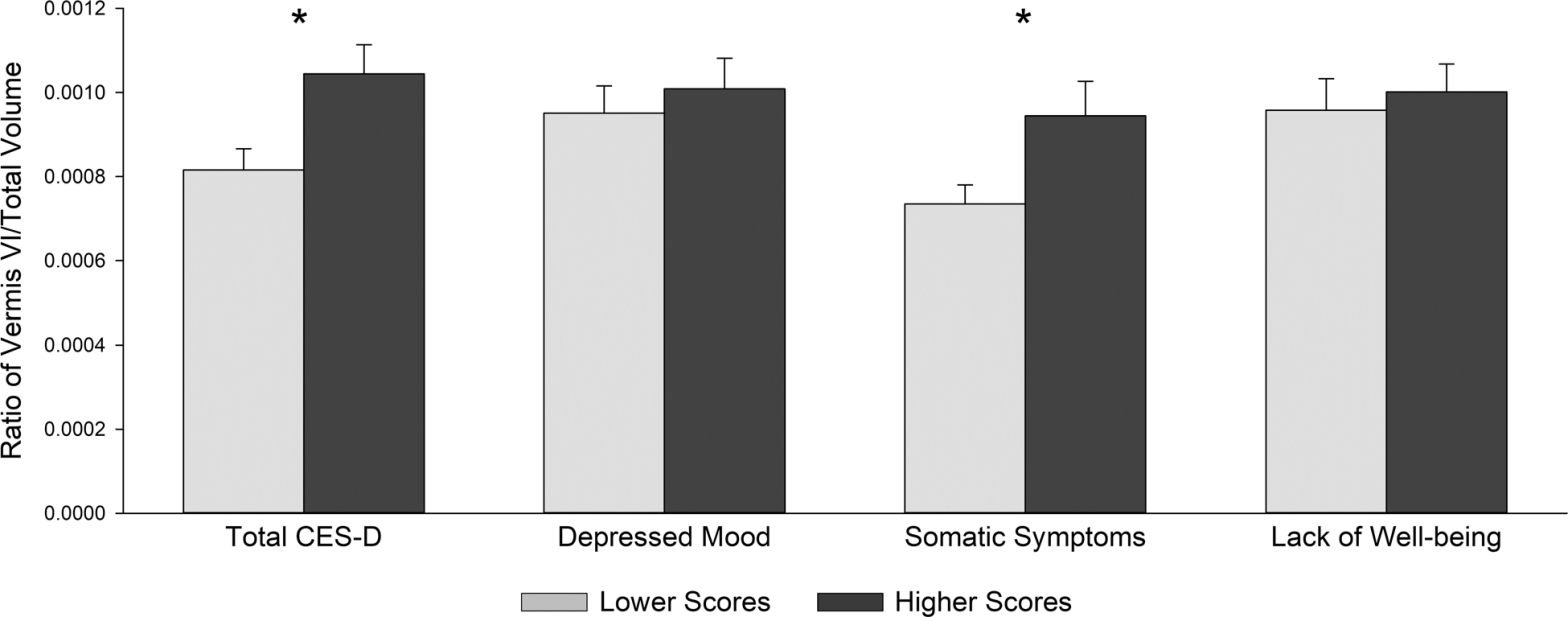
Least-square means showing the ratio of cerebellar vermis VI to total intracranial volume by Center for Epidemiologic Studies Depression Scale (CES-D) scores. Two separate regression analyses were performed for the total CES-D and for the CES-D subscales (depressed mood, somatic symptoms, and lack of positive affect subscales), which were entered simultaneously. The CES-D groups are for graphical purposes only; CES-D scores were continuous measures in all analyses. Error bars represent standard error. *p < 0.05.

**Fig. 2:**
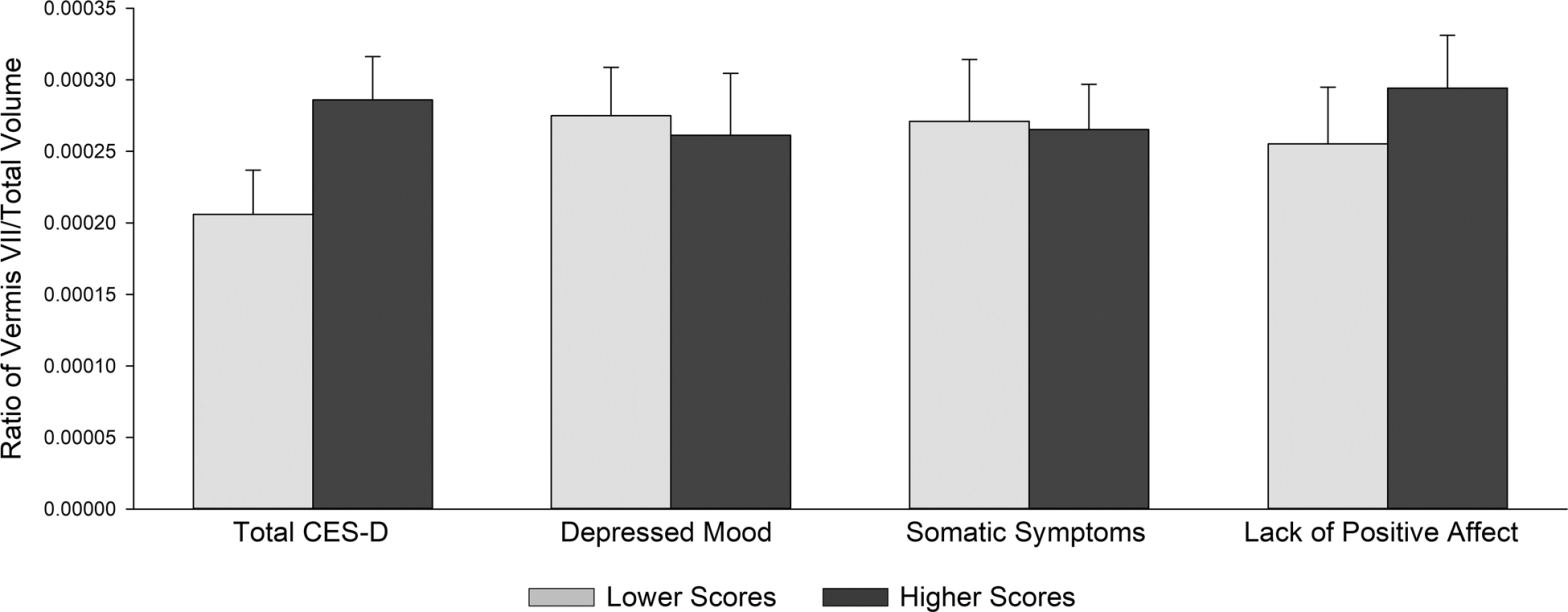
Least-square means showing the ratio of cerebellar vermis VII to total intracranial volume by Center for Epidemiologic Studies Depression Scale (CES-D) scores. Two separate regression analyses were performed for the total CES-D and for the CES-D subscales (depressed mood, somatic symptoms, and lack of positive affect subscales), which were entered simultaneously. The CES-D groups are for graphical purposes only; CES-D scores were continuous measures in all analyses. Error bars represent standard error. None of the CES-D effects were significant.

Higher total CES-D scores were associated with larger vermis VI (Fig. 1) volume (*β* = 0.378, *p* < 0.05, sr_3_^2^ = 0.139), but no significant relationship was observed between vermis VII (Fig. 2) volume and total CES-D scores (Table 2). Analysis of CES-D subscales revealed a positive relationship between somatic symptom subscale scores and vermis VI volume (*β* = 0.389, *p* < 0.05, sr_4_^2^ = 0.113). No other subscales were significantly related to vermis VI volumes. Additionally, there were no significant relationships between vermis VII volume and CES-D subscales (Table 3).

**Table 2:** Results of regression analyses: total CES-D predicting cerebellar volumes.

	Age			Sex			Total CES-D		
	*β*	*t*	*p*	*β*	*t*	*p*	*β*	*t*	*p*
Vermis VI	0.104	0.640	0.527	0.025	0.155	0.878	0.378	2.348	0.025*
Vermis VII	−0.044	−0.261	0.796	−0.053	−0.316	0.754	0.227	1.346	0.187
Vermis VIII	−0.331	−2.137	0.040*	0.017	0.113	0.910	0.276	1.793	0.082
Vermis IX	−0.208	−1.262	0.215	−0.035	−0.212	0.833	2.39	1.462	0.153
Vermis Crus I	0.176	1.025	0.313	0.126	0.743	0.463	−0.017	−0.100	0.921
Anterior CB	−0.191	−1.222	0.230	0.313	2.031	0.050*	0.210	1.357	0.184

*p ≤ 0.05. Boldface indicates statistical signifance.

**Table 3:** Results of regression analyses: CES-D subscales predicting cerebellar volumes.

	Age			Sex			Depressed mood			Somatic symptoms			Lack of positive affect		
	*β*	*t*	*p*	*β*	*t*	*p*	*β*	*t*	*p*	*β*	*t*	*p*	*β*	*t*	*p*
Vermis VI	0.070	0.409	0.686	0.045	0.278	0.783	0.040	0.213	0.833	0.389	2.094	0.044*	0.037	0.215	0.831
Vermis VII	−0.020	−0.110	0.913	−0.055	−0.313	0.756	−0.112	−0.553	0.584	0.171	0.859	0.397	0.137	0.751	0.458
Vermis VIII	−0.283	−1.924	0.063	0.029	0.208	0.836	−0.017	−0.107	0.915	−0.138	−0.866	0.393	0.527	3.614	0.001**
Vermis IX	−0.253	−1.468	0.152	−0.016	−0.099	0.922	0.082	0.433	0.668	0.297	1.595	0.121	−0.061	−0.358	0.723
Vermis Crus I	0.128	0.711	0.482	0.127	0.737	0.467	0.072	0.361	0.720	0.067	0.343	0.734	−0.216	−1.209	0.236
Anterior CB	−0.234	−1.478	0.149	0.351	2.325	0.027*	0.262	1.500	0.144	−0.059	−0.345	0.732	0.254	1.618	0.115

*p ≤ 0.05, **p ≤ 0.01. Boldface indicates statistical signifance.

*CB* cerebellum.

### Exploratory analyses

Total CES-D score did not significantly predict vermis VIII, IX, or crus I volumes (Table 2); however, we observed a positive relationship between the lack of positive affect subscale scores and vermis VIII volume (*β* = 0.527, *p* < 0.01, sr_4_^2^ = 0.248; Table 3). As expected, the anterior cerebellum was not significantly associated with CES-D total or subscale scores.

## Discussion

Over the past couple of decades, the posterior cerebellum, particularly the vermis, has been implicated in emotion processing^17,19,43^. Thus, the present study investigated the relationship between symptom dimensions of depression and subregions of the cerebellum in middle aged to older adults with SDS. Broadly, results from our analyses indicate that the relationship between regions of the vermis and depressive symptoms differs depending on the types of depressive symptoms experienced by the participant. Given these findings, our hypotheses were partially supported.

We hypothesized that overall depressive symptom severity would be negatively related to both vermal region VI and VII volumes. Additionally, we hypothesized that vermis VI and VII would have distinct relationships with symptom dimensions of depression, as measured by subscales of our depression measure. Total depressive symptoms were indeed associated with vermis VI volumes, but we observed larger rather than smaller volumes as a function of higher depressive symptoms. No relationship was found between vermis VII and total depressive symptoms. Analysis of the somatic symptoms, depressed mood, and lack of positive affect subscales suggested that this relationship was mostly driven by the somatic symptom subscale, as this was the only significant subscale in the regression model. Additionally, our exploratory analyses identified a positive relationship between vermis VIII volume and the lack of positive affect subscale scores. Thus, our hypothesis that regions of the vermis would have distinct relationships with depressive symptom subtypes was supported.

Given the consistent evidence to support the role of vermis VI in emotion processing^17,19,27,44^, it is not surprising that our analyses identified a relationship between vermis VI volume and total depressive symptom severity. Vermis VI has been found to be functionally involved in the salience network^19^, and has been hypothesized to be recruited during primary emotion processing as well^21^. In fact, Habas et al.^19^ have gone so far as to suggest that the vermis VI region of the cerebellum has a modulatory role in the subcortical aspects of the salience network, an intrinsic connectivity network disrupted in individuals with depression. The salience network is comprised of the dorsal anterior cingulate cortex, frontoinsular cortex, amygdala, and ventral striatum, and is responsible for the bottom-up detection of salient events as well as for facilitating attentional and executive resources when salient events are detected^45^. This network is important for depression because it facilitates experiences of pain and pleasure, and also determines the importance of both internal and external stimuli in order to guide behavior^46^.

Emerging evidence also suggests that a major role of the salience network is to switch between the default mode and central executive networks, and that this switching capability is impaired in individuals with MDD^45,47^. Additionally, the abnormal switching function of the salience network appears to be related to the negative response bias in depression, which has been identified as a cognitive vulnerability of depression^45^. Thus, the relationship between vermis VI and total depressive symptoms in the present study is consistent with the literature. Our additional finding that somatic symptoms were positively related to vermis VI adds to our understanding of this complex relationship between depressive symptoms and the cerebellar vermis. Given the heterogenous nature of the CES-D somatic symptoms subscale, which includes questions about apathy (e.g., “I could not get going”) and cognitive symptoms (e.g., “I had trouble keeping my mind on what I was doing”), in addition to somatic complaints (e.g., “I did not feel like eating”), more research is needed to determine vermis VI’s involvement in this particular cluster of symptoms.

The finding that the CES-D lack of positive affect subscale, which grossly measures symptoms related to anhedonia, is associated with larger vermis VIII volumes may in part be explained by vermis VIII’s functional connections to the amygdala^20,21,27^. The amygdala is intimately related to depression through its role in both the affective/frontolimbic network, which is dysregulated in late-life depression^48^, as well as its role in the reward system, which has been found to be disrupted in patients with MDD^49^. This is important, given that anhedonia can be characterized as dysfunction in the reward system leading to reduced emotional response to pleasurable events as well as a lack of engagement in goal-related behaviors^50^. In individuals with MDD, the amygdala has been shown to decrease the responsiveness of the reward (i.e., dopamine) system^51^, as well as have reduced intrinsic connectivity to the cerebellum^52^. Future research should explore the link between vermis VIII, anhedonia, and the amygdala.

While the bulk of the literature has identified smaller cerebellar volumes in relation to greater levels of depression, the finding of larger volumes in relation to higher depressive symptoms in the current study is not entirely unexpected in the context of other studies showing enlarged regional brain volumes in clinical and subthreshold depression. For example, greater severity of SDS has been associated with larger volumes in the posterior cingulate^16^, inferior temporal lobe^53^, and middle frontal gyrus^8^ among middle-aged to older adults. Additionally, depressed older adults were shown to have greater volume in the left temporal lobe, bilateral orbitofrontal cortex, and bilateral parietal cortex compared to non-depressed controls^54^ and studies in younger cohorts documented MDD-related volume enlargement in the hippocampus^55,56^. Perhaps most relevant to the current findings, a study of patients experiencing an acute major depressive episode revealed that regardless of medication status, those experiencing MDD had larger left cerebellar lobule IX volumes than controls^23^.

The significance of larger vermal volumes in relation to depressive symptoms remains unclear. Some researchers have discussed the role of “inflammaging,” or the concept that aging is characterized by chronic, low-level inflammation^57^, and believe that this could be an underlying mechanism of depression in older adults^58^. In support of this hypothesis, a meta-analysis by Dowlati et al.^59^ confirmed the presence of higher concentrations of proinflammatory cytokines in depressed participants compared to controls, which provides further evidence that the inflammatory response system is activated among depressed individuals. The researchers hypothesized that inflammation in the hippocampus in particular may be a precursor to the smaller hippocampal volumes identified in depressed individuals as the disease progresses. Other research suggests that early stages of depression are associated with increased brain volume, possibly due to the increased metabolic activity and enhanced cerebral blood flow that occurs early on in the illness, which may then be responsible for volume loss at later stages of depression^60^.

Thus, it is possible that inflammation is linked to increased brain volume seen in early stages of depression. The present study’s findings of larger vermis VI and VIII volumes in relation to higher depressive symptom load among older adults with SDS appears to provide support for this inflammation hypothesis of depression. Given that these findings were identified within a sample of adults with SDS, it is possible that our findings of larger vermis volumes, potentially due to inflammation, are precursors of the decreased volume that may occur as these individuals progress to MDD^59^. Longitudinal research is needed to identify the changes in the brain that occur as older individuals progress from SDS to MDD.

These findings become significant when examined within the context of research identifying treatment options for depression with distinct etiologies. In particular, neurobiological markers of depression have been linked to types of depressive symptoms and the causes and consequences of depression have been shown to be different based on these symptomatic and neurobiological profiles. Given the current lack of effective treatment for late-life depression, it is important to examine the different etiologies of depression in order to develop a more precise understanding of how depressive symptoms interact with the brain. The present study indicates that specific areas of the cerebellar vermis are related to distinct depressive symptoms in individuals with subthreshold symptoms. Given the heterogeneity of depressive symptomology, by identifying the neurobiological differences that underlie symptom profiles in depression, the field will become closer to developing effective, targeted treatments for individuals with depression.

The present study is not without limitations. Given the small size of the sample, the study is powered only to detect a large effect, thus, it is possible that significant relationships were not detected in our analyses. However, this also suggests that the detected effects were robust. Additionally, the sample is highly educated, primarily female, and primarily Caucasian. In this way, our sample lacks generalizability and requires replication in a larger, more diverse community sample in order to better understand the relationship between the cerebellar vermis and dimensions of depression.

## Conclusion

This study identified differential relationships between symptom dimensions of subthreshold depression and cerebellar subregion volumes. Specifically, we found positive relationships between total depressive symptom severity and vermis VI volume, somatic symptom severity and vermis VI volume, and lack of positive affect severity and vermis VIII volume. These findings highlight the role of the posterior vermis in depression. Additional studies in larger and more diverse samples will be important to better elucidate the relationship between depressive symptoms and subregions of the posterior cerebellum. Future research should also examine the posterior cerebellum as a potential treatment target in individuals with specific depressive symptom profiles.
